# Impact of the Use of Electronic Health Tools on the Psychological and Emotional Well-Being of Electronic Health Service Users (The Seventh Tromsø Study - Part 3): Population-Based Questionnaire Study

**DOI:** 10.2196/13118

**Published:** 2020-03-05

**Authors:** Andrius Budrionis, Rolf Wynn, Luis Marco-Ruiz, Kassaye Yitbarek Yigzaw, Svein Bergvik, Sunday Oluwafemi Oyeyemi, Johan Gustav Bellika

**Affiliations:** 1 Norwegian Centre for E-health Research University Hospital of North Norway Tromsø Norway; 2 Department of Clinical Medicine Faculty of Health Sciences UiT The Arctic University of Norway Tromsø Norway; 3 Division of Mental Health and Addictions University Hospital of North Norway Tromsø Norway; 4 Department of Psychology Faculty of Health Sciences UiT The Arctic University of Norway Tromsø Norway; 5 Department of Community Medicine Faculty of Health Sciences UiT The Arctic University of Norway Tromsø Norway

**Keywords:** eHealth, telemedicine, health information, Tromsø study, health-related internet use, emotions, effect, anxious, confused, knowledgeable, reassured

## Abstract

**Background:**

Electronic health (eHealth) has been described as a silver bullet for addressing how challenges of the current health care system may be solved by technological solutions in future strategies and visions for modern health care. However, the evidence of its effects on service quality and cost effectiveness remains unclear. In addition, patients’ psychological and emotional reactions to using eHealth tools are rarely addressed by the scientific literature.

**Objective:**

This study aimed to assess how the psychological and emotional well-being of eHealth service users is affected by the use of eHealth tools.

**Methods:**

We analyzed data from a population-based survey in Norway, conducted in the years 2015-2016 and representing 10,604 eHealth users aged over 40 years, to identify how the use of eHealth tools was associated with feeling anxious, confused, knowledgeable, or reassured. Associations between these four emotional outcomes and the use of four types of eHealth services (Web search engines, video search engines, health apps, and social media) were analyzed using logistic regression models.

**Results:**

The use of eHealth tools made 72.41% (6740/9308) of the participants feel more knowledgeable and 47.49% (4421/9308) of the participants feel more reassured about their health status. However, 25.69% (2392/9308) reported feeling more anxious and 27.88% (2595/9308) reported feeling more confused using eHealth tools. A high level of education and not having a full-time job were associated with positive reactions and emotions (feeling more knowledgeable and reassured), whereas low self-reported health status and not having enough friends who could provide help and support predicted negative reactions and emotions (ie, feeling anxious and confused). Overall, the positive emotional effects of eHealth use (feeling knowledgeable and reassured) were relatively more prevalent among users aged over 40 years than the negative emotional effects (ie, feeling anxious and confused). About one-fourth of eHealth users reported being more confused and anxious after using eHealth services.

**Conclusions:**

The search for health information on the internet can be motivated by a range of factors and needs (not studied in this study), and people may experience a range of reactions and feelings following health information searching on the Web. Drawing on prior studies, we categorized reactions as positive and negative reactions. Some participants had negative reactions, which is challenging to resolve and should be taken into consideration by eHealth service providers when designing services (ie, including concrete information about how users can get more help and support). There is a need for more studies examining a greater range of reactions to online health information and factors that might predict negative reactions to health information on the Web.

## Introduction

In Norway, the demand for health care services is expected to increase by 40% by 2040 [1]. Comparable estimates may be expected for other developed countries, predicting a major increase in health care budgets. Electronic health (eHealth) is often presented as a solution to this situation [2-4].

Although the pursuit of eHealth is global, evidence of its effect and impact remains conflicting and limited. It is even more difficult to find support for cost-effectiveness of eHealth tools, regardless of the claims policy makers are using to attract interest and funding for large-scale deployments [5,6]. Even though the policies may be driven by expectations rather than scientific evidence, it is easy to recognize the potential of eHealth to shape the services for the future [3]. Much can be done to enhance the experience of care, improve health of populations, and reduce per capita costs of services [7] by not only improving available technologies but also adopting and implementing novelties in the field. The use of eHealth services may increase access in remote areas and underserved populations [8-10]. However, not all population groups utilize the technology to the same degree; this has been referred to as a *digital divide* [11-15]. Recently, some studies have discussed how the consequences of internet use may differ between different groups, referred to as the *third-level digital divide* [12]. However, with some important exceptions, few have empirically studied determinants of this third-level digital divide [12,16-19]. In our study, we look at the positive and negative psychological consequences of health information seeking, which can be understood as an application of the third-level digital divide concept.

Understanding people’s reactions to eHealth services is an imperative part of evidence-based eHealth. However, the eHealth literature seems to focus more on potentially positive aspects of eHealth from the patient’s point of view, such as perceived support and increased health literacy, as major effects of eHealth tools [20-22], rather than raising critical questions. Social support is a well-established protective factor against mental health problems including anxiety, explained as a buffer against stress [23]. Many have emphasized the potentially positive effects of eHealth on shared decision making and patient empowerment [24-26]. The impact of eHealth service use on health care participation, patient involvement, and health status has been reported, whereas findings on issues such as psychological well-being, anxiety, and depression are inconsistent [20,21]. Although some of these findings were observed among patients with cancer, we may expect similar trends in other patient groups and in the general population.

Overall, eHealth seems to have a positive impact on patient-related outcomes [27,28]. However, inconsistencies and variations in the literature may relate to insufficient consideration of human factors in measures and study designs, resulting in biased findings [29]. Moreover, there has been a wide range of different methodologies used, and there is still much uncertainty with regard to what constitutes eHealth, contributing to the inconsistent findings [2].

People may search for health information online for various reasons, typically to obtain more knowledge on a specific matter [24]. However, searching and reacting to online health information cognitive processes that are also driven by emotions [30-32]. Emotions are central to the human experience and infiltrate every aspect of our existence [33]. The Information Search Process Model [34] suggests that there are six steps in information seeking and understands this process as complex and multifactorial, and it also considers emotional factors. These factors vary depending on the stage of the model from optimism to confusion and from frustration and doubt to satisfaction or disappointment [30,34,35]. This study draws on this insight into the importance of emotional factors and especially the finding that the process of information searching may increase feelings such as uncertainty [30,35]. However, we focus only on the final stage of the information searching process—that is, how people feel after having found online health information. In this study, we focus particularly on the emotional aspects and how information seekers, in retrospect, appraise the outcome of their information seeking activity. Although some may feel reassured and more knowledgeable by the information they found, others might become worried and concerned, which may lead to frustration and, perhaps, symptoms of anxiety. This may relate to a range of factors such as low income, low education, and an avoidant coping style. These attributes have been associated with vulnerability to misinformation and unfounded claims [36]. Health-related misinformation is commonly encountered on the internet [37,38]. Searching for health information may also relate to a tendency for health anxiety, defined as a concern about health in the absence of a pathology or excessive concern when there is only some degree of pathology [39]. The preoccupation with thoughts about illness in health-anxious individuals is associated with a need to search for health information [40-42]. In a study by Baumgartner and Hartmann [32], the results indicated that online health information from trustworthy websites leads to increased worries among health-anxious individuals but not among non–health-anxious individuals. Although frequent online searches for health information among health-anxious individuals were associated with an increased number of doctor appointments relating to the information found online, a negative association between online searching and doctor appointments has been found among individuals with low health anxiety, that is, frequent online health information searching among individuals with low health anxiety was found to decrease their number of doctor visits [43]. Thus, the motivation for online searching for health information and the outcome of the searching activity may be associated with feeling more knowledgeable and reassured but also with negative feelings of worries, anxiety, and frustration. Although this has been studied in limited samples [27], there is a need for large-scale studies of the relative prevalence and implications of online health-searching behavior within the general population.

In a series of four papers, we explored data on the use of eHealth related to a range of other variables that were measured in a population survey in Norway (the Tromsø Study). In part 1 [44], we presented the main findings regarding the characteristics of participants and their use of eHealth. In part 2 [45], we presented and discussed how having different illnesses influences the use of eHealth. In paper 3 (this paper), we have examined some outcomes of the use of eHealth. In part 4 [46], we studied how eHealth consumption influences actual doctor visits (KY Yigsaw, PhD, unpublished data, 2018).

Aiming to increase the understanding of the psychological effects of eHealth tools, we examined the emotions of respondents who had used eHealth tools to obtain health information. Specifically, we examined the positive emotions of feeling more knowledgeable and reassured and the negative emotions of feeling anxious and confused. On the basis of existing literature, we hypothesized that the positive reactions, in general, would be more prevalent than the negative reactions.

## Methods

### Overview

To research the influence of eHealth use on the respondents’ emotional state, data from the seventh survey of the Tromsø Study (Tromsø 7) population-based study were analyzed. The Tromsø Study is a representative survey collecting a wide range of data from the population in the municipality of Tromsø in Northern Norway [47]. In the seventh version of the study, people aged over 40 years were included, and data on the use of eHealth services were collected for the first time.

The survey was conducted in the years 2015-2016 and included 21,083 participants in the overall study. A total of 10,604 respondents reported at least one single use of the internet service (Web search engines, video search engines, health apps, and social media) for searching health information during the last year [44]. Only these internet service users were included in this study.

### Dependent Variables

The dependent variables studied in this paper were participants’ responses to questions regarding whether they felt either anxious, confused, more knowledgeable, or more reassured after using eHealth tools (Web search engines, video search engines, health apps, and social media). Responses were provided on a Likert scale format with the values *never*, *once*, *a few times*, and *often*. For the analysis, these were recoded into binary variables indicating *never* or *once or more* (collapsing the *once*, *few times*, and *often* categories).

### Independent Variables

Demographic variables such as age, sex, self-reported health status, education, household income, and occupation were included in the analyses. Age was recoded into an ordinal variable of four age groups (40-49, 50-59, 60-69, and ≥70 years). The occupation groups *Disability benefits receivers* and *Family income supplement receivers* were joined into a single group titled *Social benefits receivers*. It resulted in a total of seven occupation groups used in the analysis (*Works full-time*, *Works part-time*, *Unemployed*, *Housekeeping*, *Retired*, *Student or military service*, and *Social benefits receivers*). Initial household income groups (<150,000 kr / <15,963 US, 150,000-250,000 kr / 15,963-26,605 US, 251,000-350,000 kr / 26,712-37,247 US, 351,000-550,000 kr / 37,354-58,532 US, 551,000-750,000 kr / 58,638-79,816 US, 751,000-1,000,000 kr / 79,923-106,422 US, and >1,000,000 kr / >106,422 US) were reorganized to obtain a better balance in the number of participants per group (0-250,000 kr / 0-26,605 US, 251,000-450,000 kr / 26,712-47,890 US, 451,000-750,000 kr / 47,996-79,816 US, 751,000-1,000,000 kr / 79,923-106,422 US, and >1,000,000 kr / >106,422 US). No preprocessing was applied to sex, self-reported health status, and education variables.

Additional variables were included, such as respondents living with a spouse, having enough friends who could provide help and support, and having enough friends to talk confidentially with. These variables were coded in a binary form (*Yes* or *No*).

Current or past medical conditions (high blood pressure, heart attack, heart failure, atrial fibrillation, angina pectoris, stroke, diabetes, kidney diseases, bronchitis, asthma, cancer, rheumatoid arthritis, arthrosis, migraine, psychological problems, and chronic pain) were also included in the analyses. The medical condition variables were converted into a single binary variable, indicating the existence of at least one condition currently or in the past. A detailed analysis of Tromsø 7 data with regard to medical conditions and eHealth use is presented in part 2 of this paper series [45].

All independent variables are summarized in Table 1.

**Table 1:** Characteristics of the study sample (N=9308).

Variables		Values, n (%)
**Age group (years)**		
	40-49	3601 (38.68)
	50-59	2595 (27.88)
	60-69	2427 (26.07)
	≥70	685 (7.36)
**Gender**		
	Women	5213 (56.01)
	Men	4095 (43.99)
**Self-reported health status**		
	Very bad	28 (0.30)
	Bad	504 (5.41)
	Neither good nor bad	2124 (22.82)
	Good	5164 (55.48)
	Excellent	1488 (15.99)
**Education**		
	Primary/partly secondary	1056 (11.35)
	Upper secondary	2361 (25.37)
	Tertiary short (<4 years of college)	2104 (22.60)
	Tertiary long (≥4 years of college)	3787 (40.69)
**Occupation**		
	Full-time worker	6495 (69.78)
	Part-time worker	819 (8.80)
	Unemployed	73 (0.78)
	Housekeeping	33 (0.35)
	Retired	1049 (11.27)
	Student or military service	37 (0.40)
	Social benefits receiver	802 (8.62)
**Household income (kr/US)^a^**		
	0-250,000 kr / 0-26,605 US	204 (2.19)
	251,000-450,000 kr / 26,712-47,890 US	1039 (11.17)
	451,000-750,000 kr / 47,996-79,816 US	2463 (26.46)
	751,000-1,000,000 kr / 79,923-106,422 US	2559 (27.49)
	>1,000,000 kr / >106,422 US	3043 (32.69)
**Lives with a spouse**		
	No	1989 (21.37)
	Yes	7319 (78.63)
**Has enough friends to talk confidentially with**		
	No	1298 (13.94)
	Yes	8010 (86.14)
**Has enough friends who could give help and support**		
	No	1028 (11.04)
	Yes	8280 (88.96)
**Medical condition currently or in the past**		
	No	2442 (26.24)
	Yes	6866 (73.76)

^a^Norwegian kroner (kr) / American dollar (US).

### Data Analysis

The removal of cases containing missing values in the dependent or independent variables resulted in a complete dataset of N=9308 included in the further analyses. Data were analyzed with the IBM SPSS Statistics for Macintosh, Version 25.0 (IBM Corp, Armonk, NY). Descriptive statistics were used for data exploration, whereas associations between dependent and independent variables were analyzed using a logistic regression model. Goodness of fit was assessed using a Holsmer-Lemeshow test, and only statistically significant models are reported.

The following interactions between variables were tested in all four logistic regression models: age group and self-reported health status; occupation and household income; education and household income; age group and household income; age group and any medical condition; and having enough friends who could provide help and support and having enough friends to talk confidentially with.

Analyses were run including all independent variables into the regression models. Interaction terms that were considered important on the basis of the domain knowledge or that could act as confounders were also included. The interactions were tested before the regression analyses. Only statistically significant variables are reported.

### Ethics

The Regional Ethical Committee for Medical and Health Research Ethics approved Tromsø 7 (REK Nord, reference 2014/940). All participants provided written consent.

## Results

### Descriptive Analysis

**Figure 1:**
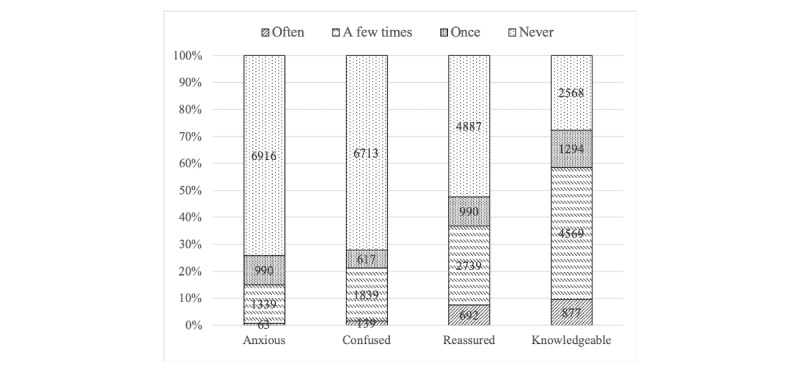
Distribution of dependent variables (N=9308).

Looking at the distribution of the dependent variables (Figure 1), it is evident that the use of eHealth tools is more often associated with positive than negative feelings. Almost half of the users (4421/9308, 47.49%) reported feeling reassured and close to three-quarters (6740/9308, 72.41%) reported feeling more knowledgeable, compared with less than one-third of the respondents who reported becoming anxious (2392/9308, 25.69%) or confused (2595/9308, 27.88%) after using eHealth tools. At the same time, more than two-thirds of the participants reported no anxiety (6916/9308, 74.31%) or confusion (6713/9308, 72.12%) associated with their use of eHealth tools, whereas the ratio of respondents who had never felt reassured or more knowledgeable was 52.51% (4887/9308) and 27.59% (2468/9308), respectively.

### Relation Between Dependent and Independent Variables

#### Feeling Anxious

We conducted a logistic regression analysis with the dependent variable *feeling anxious* following the use of eHealth tools. The following predictors were included: age group, sex, self-reported health status, education, occupation, household income, lives with a spouse, has enough friends to talk confidentially with, has enough friends who could give help and support, and medical condition currently or in the past. The logistic regression model fitted well with data (Hosmer-Lemeshow goodness-of-fit chi-square=6.949, *P*=.54). The full model was statistically significant (χ^2^_5_=2676.6; *P*<.001). None of the interactions were significant.

Seven variables made unique independent contributions to the model. One variable had a significant positive contribution to feeling anxious after using eHealth tools. Participants having a medical condition currently or in the past had relatively higher odds (odds ratio [OR] 1.239) to feel anxiety than the participants without any disease (Table 2).

**Table 2:** Significant contributions to feeling anxious after using electronic health tools.

Significant variables^a^		Odds ratio (95% CI)	*P* value
**Age group (years)**			
	40-49	1.00	Ref^b^
	50-59	0.818 (0.728-0.920)	.001
	60-69	0.679 (0.595-0.776)	<.001
	≥70	0.699 (0.610-0.746)	.02
**Sex**			
	Women	1.00	Ref
	Men	0.674 (0.610-0.746)	<.001
**Self-reported health status**			
	Very bad	1.00	Ref
	Excellent	0.606 (0.417-0.879)	.008
**Occupation**			
	Full-time worker	1.00	Ref
	Retired	0.760 (0.586-0.985)	.04
**Household income (1000 kr)**			
	0-250	1.00	Ref
	251-450	0.719 (0.524-0.986)	.04
	751-1000	0.666 (0.476-0.932)	.02
	>1000	0.633 (0.447-0.895)	.01
**Enough friends who could provide help and support**			
	No	1.00	Ref
	Yes	0.662 (0.554-0.791)	<.001
**Medical condition currently or in the past**			
	No	1.00	Ref
	Yes	1.239 (1.099-1.396)	<.001

^a^Complete list of variables included in the model: age group (40-49, 50-59, 60-69, and ≥70), sex (women and men), self-reported health status (very bad, bad, neither good nor bad, good, and excellent), education (primary/partly secondary, upper secondary, tertiary short, and tertiary long), occupation (works full-time, works part-time, unemployed, housekeeping, retired, student or military service, and social benefits receiver), household income (0-250,000 kr / 0-26,605 US, 251,000-450,000 kr / 26,712-47,890 US, 451,000-750,000 kr / 47,996-79,816 US, 751,000-1,000,000 kr / 79,923-106,422 US, and >1,000,000 kr / >106,422 US), lives with a spouse (no and yes), has enough friends to talk confidentially with (no and yes), has enough friends who could give help and support (no and yes), and medical condition currently or in the past (no and yes).

^b^Ref: reference group.

In total, six independent variables contributed negatively to feeling anxious after using eHealth tools. The OR of feeling anxious decreased with age; a few cases of anxiety were observed among participants older than 60 years. Men were less likely (OR 0.674) to feel anxious than women. Participants who rated their own health as excellent had lower odds (OR 0.606) of feeling anxious than those rating their health as very bad. Retired participants were less likely (OR 0.760) to feel anxious than full-time employees. Higher income was associated with a lower chance of anxiety. The tendency to become anxious was the least prevalent in the tow highest income groups (OR 0.666 and OR 0.633). Participants who had enough friends that could provide help and support had lower odds (OR 0.662) of feeling anxious after using eHealth tools. Significant predictors of feeling anxious among eHealth users are summarized in Table 2.

To sum up, those who were more likely to become anxious after using eHealth tools tended to be younger women rating their own health lower than *excellent*, currently or previously suffering from a medical condition, and not having enough friends who could provide help and support. They were likely in a lower household income group and were not retired.

#### Feeling Confused

We performed a logistic regression analysis with the dependent variable *feeling confused* following the use of eHealth tools. The following predictors were included: age group, sex, self-reported health status, education, occupation, household income, lives with a spouse, has enough friends to talk confidentially with, has enough friends who could give help and support, and medical condition currently or in the past. The logistic regression model fitted well with data (Hosmer-Lemeshow goodness-of-fit chi-square=6.376, *P*=.61). The full model was statistically significant (χ^2^_6_=2220.5; *P*<.001). The influence of the independent variables on feeling confused after using eHealth tools is summarized in Table 3.

A total of five variables made unique independent contributions to the model. Having a current or previous medical condition had a positive contribution to feeling confused. Participants who had one or more medical conditions had higher odds (OR 1.16) of feeling confused after using eHealth tools in comparison with the healthy individuals.

In total, four variables contributed negatively to confusion after using eHealth tools. Increase in age contributed negatively to feeling confused, and the level of confusion was relatively lower in age groups 3 and 4 (≥60 years). Women were more likely to feel confused than men (OR 0.694). Participants who rated their health as *good* or *excellent* were less likely (OR 0.696 and OR 0.541, respectively) to feel confused than with the ones rating their health as *very bad*. The retired had lower odds (OR 0.769) of feeling confused than full-time workers.

In summary, the use of eHealth tools was more likely to cause confusion in younger women who currently or previously were had any medical condition. They were more likely to have relatively poor self-rated health status and were less likely to be retired.

A significant interaction was observed between the variables having enough friends who could provide help and support and having enough friends to talk confidentially with (OR 0.690, 95% CI 0.488-0.976; *P*=.04). This shows that the odds for becoming confused were lower for those reporting both enough friends to talk confidentially with and who could provide help and support (OR 0.690) compared with those who only responded yes on one of these questions.

**Table 3:** Significant contributions to feeling confused after using electronic health tools.

Significant variables^a^		Odds ratio (95% CI)	*P* value
**Age group (years)**			
	40-49	1.00	Ref^b^
	50-59	0.782 (0.698-0.875)	<.001
	60-69	0.612 (0.537-0.697)	<.001
	≥70	0.703 (0.520-0.951)	.02
**Sex**			
	Women	1.00	Ref
	Men	0.694 (0.629-0.766)	<.001
**Self-reported health status**			
	Very bad	1.00	Ref
	Good	0.696 (0.489-0.989)	.04
	Excellent	0.541 (0.374-0.782)	.001
**Occupation**			
	Full-time worker	1.00	Ref
	Retired	0.769 (0.596-0.992)	.04
**Medical condition currently or in the past**			
	No	1.00	Ref
	Yes	1.16 (1.035-1.302)	.01
Significant interaction observed between variables having enough friends who could give help and support and enough friends to talk confidentially with		0.690 (0.488-0.976)	.04

^a^The complete list of variables included in the model is as follows: age group (40-49, 50-59, 60-69, and ≥70), sex (women and men), self-reported health status (very bad, bad, neither good nor bad, good, and excellent), education (primary/partly secondary, upper secondary, tertiary short, and tertiary long), occupation (works full-time, works part-time, unemployed, housekeeping, retired, student or military service, and social benefits receiver), household income (0-250,000 kr / 0-26,605 US, 251,000-450,000 kr / 26,712-47,890 US, 451,000-750,000 kr / 47,996-79,816 US, 751,000-1,000,000 kr / 79,923-106,422 US, and >1,000,000 kr / >106,422 US), lives with a spouse (no and yes), has enough friends to talk confidentially with (no and yes), has enough friends who could give help and support (no and yes), and medical condition currently or in the past (no and yes).

^b^Ref: reference group.

#### Feeling Knowledgeable

We conducted a logistic regression analysis with the dependent variable *feeling knowledgeable* following the use of eHealth tools. The following predictors were included: age group, sex, self-reported health status, education, occupation, household income, lives with a spouse, has enough friends to talk confidentially with, has enough friends who could give help and support, and medical condition currently or in the past. The logistic regression model fitted well with data (the Hosmer-Lemeshow goodness-of-fit chi-square=2.661, *P*=.95). The full model was statistically significant (χ^2^_6_=2254.7; *P*<.001). The influence of the independent variables on feeling more knowledgeable after eHealth use is summarized in Table 4.

A total of four variables made unique independent contributions to the model. The level of education contributed positively to feeling more knowledgeable. A higher level of education was associated with higher odds for feeling more knowledgeable after using eHealth tools: Participants who had upper secondary education (OR 1.601), short university education (OR 2.526), and long university education (OR 2.858) had higher odds of feeling more knowledgeable in comparison with individuals who had primary or partly secondary education (Table 4). Having a medical condition currently or in the past had a significant positive contribution to feeling more knowledgeable. Participants who had any of the earlier mentioned conditions were more likely (OR 1.119) to feel more knowledgeable after using eHealth tools. The occupation variable contributed positively to feeling knowledgeable. Part-time workers (OR 1.211), unemployed (OR 2.429), and social benefits receivers (OR 1.448) had higher odds of feeling more knowledgeable than full-time workers.

Age group 3 (participants aged 60-69 years) had significantly lower odds (OR 0.787) of feeling knowledgeable than age group 1 (individuals aged 40-49 years).

In summary, a typical eHealth user who felt more knowledgeable after using eHealth tools was a younger person (no significant gender differences) with higher education, who currently had a medical condition or had a history of a medical condition. He/she worked part-time, was unemployed, or received social benefits.

A significant interaction was observed between the variables having enough friends who could give help and support and having enough friends to talk confidentially with (OR 0.631, 95% CI 0.427-0.933; *P*=.021). This shows that the odds of having enough friends to talk confidentially with were lower (OR 0.631) in participants who had enough friends who could provide help and support in comparison with the ones who did not.

**Table 4:** Significant contributions to feeling more knowledgeable after using electronic health tools.

Significant variables^a^		Odds ratio (95% CI)	*P* value
**Age group (years)**			
	40-49	1.00	Ref^b^
	60-69	0.787 (0.693-0.893)	<.001
**Education**			
	Primary	1.00	Ref
	Upper secondary	1.601 (1.375-1.865)	<.001
	Tertiary short^c^	2.526 (2.142-2.980)	<.001
	Tertiary long^d^	2.858 (2.437-3.351)	<.001
**Occupation**			
	Full-time worker	1.00	Ref
	Part-time worker	1.211 (1.015-1.444)	.03
	Unemployed	2.429 (1.263-4.672)	.008
	Social benefits receiver	1.448 (1.193-1.759)	<.001
**Medical condition currently or in the past**			
	No	1.00	Ref
	Yes	1.119 (1.001-1.250)	.047
Significant interaction observed between variables has enough friends who could give help and support and has enough friends to talk confidentially with		0.631 (0.427-0.933)	.02

^a^The complete list of variables included in the model is as follows: age group (40-49, 50-59, 60-69, and ≥70), sex (women and men), self-reported health status (very bad, bad, neither good nor bad, good, and excellent), education (primary/partly secondary, upper secondary, tertiary short, and tertiary long), occupation (works full-time, works part-time, unemployed, housekeeping, retired, student or military service, and social benefits receiver), household income (0-250,000 kr / 0-26,605 US, 251,000-450,000 kr / 26,712-47,890 US, 451,000-750,000 kr / 47,996-79,816 US, 751,000-1,000,000 kr / 79,923-106,422 US, and >1,000,000 kr / >106,422 US), lives with a spouse (no and yes), has enough friends to talk confidentially with (no and yes), has enough friends who could give help and support (no and yes), and medical condition currently or in the past (no and yes).

^b^Ref: reference group.

^c^College/university, <4 years.

^d^College/university, ≥4 years.

#### Feeling Reassured

We performed a logistic regression analysis with the dependent variable *feeling reassured* following the use of eHealth tools. The following predictors were included: age group, sex, self-reported health status, education, occupation, household income, lives with a spouse, has enough friends to talk confidentially with, has enough friends who could give help and support, and medical condition currently or in the past. The logistic regression model fitted well with data (Hosmer-Lemeshow goodness-of-fit chi-square=4.953, *P*=.76). The full model was statistically significant (χ^2^_5_=191.7; *P*<.001). None of the interactions were significant. The influence of the independent variables on feeling more reassured after using eHealth tools use is summarized in Table 5.

A total of four variables made unique independent contributions to the model. Education contributed positively to feeling reassured after using eHealth tools. The largest effect size was observed in the short university/collage education group (OR 1.339). The occupation variable had a positive contribution to feeling reassured. Unemployed participants and social benefits receivers were groups that differed the most from other participants. The OR of an unemployed person feeling reassured after using eHealth tools was 1.67, whereas for receivers of disability benefits, it was 1.22 in comparison with full-time employees (Table 5).

The age group and gender variables contributed negatively to feeling reassured after using eHealth tools. Men were less likely (OR 0.657) to feel reassured after using eHealth tools in comparison with women. The biggest difference with regard to age was between groups 1 (40-49 years) and 3 (60-69 years; OR 0.779).

In short, those who were more likely to feel reassured after using eHealth tools were the young, female, highly educated, unemployed, or receiving social benefits.

**Table 5:** Significant contributions to feeling reassured after using electronic health tools.

Significant variables^a^		Odds ratio (95% CI)	*P* value
**Age group (years)**			
	40-49	1.00	Ref^b^
	60-69	0.779 (0.695-0.873)	<.001
**Sex**			
	Women	1.00	Ref
	Men	0.657 (0.603-0.717)	<.001
**Education**			
	Primary	1.00	Ref
	Upper secondary	1.179 (1.016-1.369)	.03
	Tertiary short^c^	1.339 (1.147-1.565)	<.001
	Tertiary long^d^	1.192 (1.026-1.385)	.02
**Occupation**			
	Full-time worker	1.00	Ref
	Unemployed	1.673 (1.040-2.690)	.03
	Social benefits receiver	1.220 (1.029-1.446)	.02

^a^The complete list of variables included in the model is as follows: age group (40-49, 50-59, 60-69, and ≥70), sex (women and men), self-reported health status (very bad, bad, neither good nor bad, good, and excellent), education (primary/partly secondary, upper secondary, tertiary short, and tertiary long), occupation (works full-time, works part-time, unemployed, housekeeping, retired, student or military service, and social benefits receiver), household income (0-250,000 kr / 0-26,605 US, 251,000-450,000 kr / 26,712-47,890 US, 451,000-750,000 kr / 47,996-79,816 US, 751,000-1,000,000 kr / 79,923-106,422 US, and >1,000,000 kr / >106,422 US), lives with a spouse (no and yes), has enough friends to talk confidentially with (no and yes), has enough friends who could give help and support (no and yes), and medical condition currently or in the past (no and yes).

^b^Ref: reference group.

^c^College/university, <4 years.

^d^College/university, ≥4 years.

#### Summary of the Findings

Our findings on the psychological effects of eHealth tools are consistent with previous studies performed in several European countries [27,28] and are summarized in Table 6.

**Table 6:** Summary of findings (significant predictors).

Significant variables	Feeling reassured and feeling more knowledgeable	Feeling anxious and feeling confused
Gender (men and women)	Women (reassured), both genders (more knowledgeable)	Women
Age group (years; 40-49, 50-59, 60-69, and ≥70 years)	Younger	Younger
Self-rated health status (very bad, bad, neither good nor bad, good, and excellent)	Not significant	Below average (neutral, bad, and very bad)
Education (primary/partly secondary, upper secondary, tertiary short, and tertiary long)	High level of education	Not significant
Employment (works full-time, works part-time, unemployed, housekeeping, retired, student or military service, and social benefits receiver)	Part-time, unemployed, student or military service, or receives social benefits	Not retired
Medical condition currently or in the past (yes and no)	Yes	Yes
Enough friends, who could provide help and support (yes and no)	Not significant	No

## Discussion

### Principal Findings

Despite the global trends of accelerating technological developments and a rapid implementation of digitalized care processes in health systems, few studies have investigated how users feel and react after health information searching. The results of this study shed some light on an area of knowledge that has remained unclear owing to contradictory results of previous research [20,21]. Illness may have a huge impact on people’s lives, including on well-being and other psychological outcomes. It is imperative that patients are informed in a clear and balanced way, so that they can play an active and constructive role in the management of their illness. Inaccurate, imbalanced, or misleading information can generate confusion and lead patients to wrong choices [16,17].

Kuhlthau’s theory related to information searching points out that information searching is informed by emotions and cognitive capabilities [30,35,36]. In our study, we have drawn on Kuhlthau’s insight and focused on the final part of the information search process and the emotional reactions to online health information. We found that while most had positive reactions following health information searching, about a quarter had negative reactions. How people react to health information will be determined by a range of factors, including cognitive and emotional factors, and people with health anxiety are in general likely to respond more negatively than others [32]. However, online health information may be designed in such a way that it may reduce some of the stress and negative emotions related to the information search process itself [31]. Considering our finding that approximately a quarter of participants experienced negative feelings after health information searching, it might, for instance, be helpful and reduce stress and negative feelings if provider/institution names and contact information were systematically provided together with the online health information.

The psychological effects of health information will vary and some information is likely to produce stronger emotional reactions than other types, such as information about life-threatening diseases [48]. Information provision is the main purpose of many eHealth services, based on the assumption that successful eHealth services may increase knowledge on health issues among their users. We examined some reactions to the use of eHealth services, and our results confirmed that the majority of users felt more knowledgeable and reassured after using eHealth services. In this section, findings from this study are discussed in the light of previous studies reporting relevant results.

The eHealth Trends Study [27], a telephone survey of 7903 respondents from Norway, Denmark, Germany, Greece, Poland, Portugal, and Latvia in 2005, found that 30% of internet users felt reassured or relieved after searching for information about health or illness online, whereas only 15% reported feeling anxious. In a subset of the general Norwegian population data [49], almost a quarter of the users (23%) reported feeling reassured by online health information, whereas 10% reported increased anxiety from the same type of information. The relative numbers of the participants who felt reassured and anxious doubled (42.6% and 17.3%, respectively), if only the eHealth user population was considered [50]. In our study, performed 10 years later, we found a slightly higher percentage of those feeling reassured (47.5%) and those feeling anxious (25.7%) among the eHealth users. Thus, the findings in our study are consistent with previous studies.

Medlock et al [28] reported similar findings in their small sample study from 2011 of Dutch seniors (aged 49-94 years; N=100; 85% older than 65 years). Feelings of anxiety (38%) and confusion (39%) were relatively higher than the figures in our study (25.7% and 27.9%, respectively), whereas more comparable figures were reported on feeling reassured in 56% (47.5% in Tromsø 7) and feeling more knowledgeable in 69% (72.4% in Tromsø 7). We may only speculate on the reasons for these differences, as the timing, the population, and the design of the studies vary. A stereotypical assumption that users of higher age, as in the Dutch study, would be more easily confused or worried by eHealth services is not confirmed by our data. On the contrary, tendencies of confusion and anxiety diminished by increasing age. However, the older age groups felt that they tended to be less reassured and less knowledgeable.

Several publications emanating from the US Health Information National Trends Survey have examined associations between online health information and participants’ knowledge and health behavior [16-19]. Although these have not centered primarily on emotional reactions, they show that there are important differences in how participants respond to online health information, supporting the concept of a *third-level digital divide*. In our study, we draw on the concept of the *third-level digital divide* and demonstrate empirically that the concept is relevant also for psychological reactions and emotions.

In our study, seven variables made significant contributions in predicting individual feelings after using eHealth tools (Table 6). Female gender (except feeling more knowledgeable) and younger age were associated with both positive and negative feelings. This is supported by earlier studies, showing that women are generally more engaged in eHealth activities [27,51], thus larger effects, both positive and negative, can be expected among women. Gender differences are also well documented in mental health issues, with reports of more worries and concerns and higher rates of anxiety and depression in women, compared with men [52]. The reduced anxiety and confusion among older users may reflect general age differences in perceptions and worries of health issues. Epidemiological studies report a general tendency of reduced anxiety with increasing age [53]. In our study, increasing age contributed negatively to both positive and negative feelings. On the basis of our data, we cannot conclude, but may speculate, if this may be related to decreasing levels of computer literacy in older participant groups, which may lower the expectations for eHealth tools and, consequently, the probability of both positive and negative effects. It may also be that older participants in general have more experience and thereby have developed coping strategies that make them less prone to be influenced emotionally by online health information.

Chronic illness is common among adults in the Western countries, and 45% of US adults have at least one chronic illness [54]. Having a current or past medical condition predicted both increased positive and negative emotions following eHealth use. One way to understand this finding is that the stakes can be higher for people who have (or have had) a medical condition. They have a personal experience of being ill and might be searching for information pertaining directly to themselves, which might make the health information feel more important or relevant to them than people who are completely healthy and well. Consequently, for people with a medical condition, information that is perceived as negative might result in stronger negative emotions and information that is perceived as positive might result in stronger positive reactions. Some of those who state that they have (or have had) a medical condition suffer from psychological problems, including different forms of anxiety. Prior research has suggested that people with health anxiety react stronger and more negatively to health information than others [32,43].

Socioeconomic status (SES) is a well-known and central indicator of health and use of health services, and low scores on SES indicators such as educational level, household income, and work status have been associated with relatively poorer health and higher use of certain health services [55,56]. However, in Norway, the use of specialist services has been higher among the more educated [57]. A detailed analysis of Tromsø 7 data with regard to health care services consumption and eHealth use is presented in part 4 of this paper series.

Our results confirm that feelings and reactions after using eHealth services are associated with SES and also show some interesting patterns that can be considered to reflect the *third-level digital divide*, that is, different groups of users have different emotional reactions to the use of the services [11,12]. Although the negative reaction of feeling anxious is associated with lower income, the positive reactions of feeling reassured and more knowledgeable are both associated with higher education. In general, anxiety and depression are more prevalent in lower-income populations [58], and this was also reflected in our results. We may speculate whether this indicates that higher education may provide individuals with cognitive tools to process health information, and perhaps critically judge the credibility of the information and the source of information. As an indicator of SES, education may reflect being more resourceful. Thus, the effect of education may relate to the group of higher educated participants tending to be more resourceful and may more easily navigate in the heath care system and get access to specialized health services, compared with the group of lower educated participants. Indeed, previous research indicates that the educational level is a significant predictor of health disparities and that health literacy partly mediates this relation [59,60].

Another interesting finding is the importance of social support on feeling anxious following the use of eHealth services. Notably, there was a significant effect of the interaction between having enough friends, expected to provide help and support, and having enough friends to talk confidentially with on feeling confused after using eHealth services. Our data suggest that talking to friends who they perceive as supportive may protect them from being confused by their use of eHealth services. This supports what has been established in the research on social support, that having a social network or talking to friends is not in itself sufficient to gain an effect of social support. The effect is seen when social networks are perceived as supportive [61].

### Limitations

The population of the Tromsø 7 participants originates from the city of Tromsø in the North of Norway. Although the Tromsø 7 population represents Tromsø well, it may not be completely representative to other geographical locations.

The limitations of establishing any causality between the use of eHealth resources and feeling anxious, confused, reassured, or more knowledgeable is a general weakness of cross-sectional design studies. Owing to the design of the study and the questionnaire, we cannot draw any conclusions on the effect of eHealth tools on the positive and negative feelings in users. The findings are merely associations, and we cannot tell whether the use of eHealth tools caused any changes in the positive and negative health-related feelings users may have had beforehand. Results discussed in this paper present the status of feelings in eHealth users; however, this status may not be directly attributed to the use of eHealth. Additional measures, such as health anxiety, personality profiles, or coping styles or strategies, may have contributed to the interpretation of the findings, but were not available for this study.

Although we have included a range of highly relevant variables, we lack some variables that might be relevant, such as previous experience with health services, technological literacy, and psychological factors such as personality, cognitive capacity, coping strategies, self-efficacy, and tendencies to worrying, anxiousness, and depression. We also lack a broader range of variables on social support and variables that could give even more nuanced information about the importance of partners to the reactions to the use of eHealth tools. Future studies should consider including some of these variables. Our study underlines the importance of including appraisals and emotional reactions to the information seeking process. However, as we only have retrospective data on emotional reactions to the health information–seeking process, we are unable to link our results to the information seeking process itself.

### Conclusions

This study demonstrated that positive (reassured and more knowledgeable) feelings after using eHealth services are approximately 2.2 times more common than negative (anxious and confused) feelings in eHealth users. Women have stronger effects than men, and younger users are more likely to have both positive and negative effects than the older ones.

A person, who is likely to have positive outcomes of eHealth, is highly educated, but not in full-time employment. He/she is likely experiencing a medical condition currently or has experienced one in the past. Negative outcomes are more common in people with a poorer self-reported health condition; these people are also likely experiencing a medical condition currently or have experienced one in the past. They are likely not retired and do not have enough friends who could provide help and support.

Online health searching may be motivated by many different factors and needs that we have not studied in this work. Furthermore, people may experience different reactions and emotions when they have searched for health information online. In our study, drawing on prior literature, we have classified reactions into *positive* and *negative*. It is important that some participants reacted negatively, and this issue should be addressed by eHealth service providers when designing services—for instance, by including concrete information about how users can get more help and support. More studies that examine a greater range of reactions to online health information are needed, as are studies that examine more detailed factors that might predict negative reactions to online health information.

## Abbreviations

eHealth: electronic health
OR: odds ratio
SES: socioeconomic status
Tromsø 7: seventh survey of the Tromsø Study
